# Thrust system, flower structures and transpressive duplexes in Zeidun-Kareim Belt, Central Tectonic Province, Egyptian Nubian Shield (East African Orogen)

**DOI:** 10.1038/s41598-024-79864-4

**Published:** 2024-11-21

**Authors:** Zakaria Hamimi, Magdi Khalil, Hoda Ragab Saad, Mahmoud N. El-Tawapty, Hosam Khamis, Ahmed Elbahrawy, Ashraf S. Abdelmaksoud

**Affiliations:** 1 Geology Department, Faculty of Science, Benha University, Benha, 13518 Egypt; 2 Geology Department, Faculty of Science, Damietta University, New Damietta, 34517 Egypt; 3 Nuclear Material Authority, Cairo, Egypt; 4 Geology Department, Faculty of Science, Menoufia University, Shiben El Kom, 51123 Egypt

**Keywords:** Arabian-Nubian Shield, East African Orogen, Najd Orogeny, Thrust duplexes, Transpression, Geodynamics, Geology, Tectonics

## Abstract

In this study, we explore thrust system, flower structures and transpressive duplexes in the Zeidun-Kareim belt (ZKB) in the Egyptian Nubian Shield (ENS; northwestern ANS). Filed observations and the measured stretched lineations along thrust planes reveal two main thrusting directions; ESE- (to NE- and NNE-)- and NW- (to WNW-)-directions belonging to two main phases of contraction. The timing of both phases is indirectly constrained. The older ESE- (to NE- and NNE-)-vergent thrusting is attributed to the E-W Gondwana assembly. The younger NW- (to WNW-)-vergent thrusting is akin to the Najd Orogeny. The poles to the in-sequence thrusts lie close to the poles of stretching lineations. The mean orientations of thrust propagation are, respectively, 059° and 309°. Propagation of thrusting along the two main thrusting directions resulted in the formation of a complete geometry of thrust duplex system, imbricate nappe stacking, flower structures and thrust-related folding. The top-to- ESE- (to NE- and NNE-) transpression reflects dextral sense, whereas the top-to- NW- (to WNW-) transpression exhibits sinistral sense, in compatible with those recorded and argued by many authors elsewhere in the ENS and the entire ANS. Our study fully constraints the ZKB spathio-temporal tectonic evolution which involves three main stages.

## Introduction

The Egyptian Nubian Shield (ENS) is one of the tectonostratigraphic terranes of the Arabian-Nubian Shield (ANS). The latter was regarded by many workers to represent the low grade upper crustal equivalent of the Mozambique Belt (MB) where both ANS and MB form the East African Orogen (EAO); a conspicuous mountaineous chain extending in East Africa for southends of kilometers. The geology and tectonic evolution of the ANS and the EAO have been discussed in numerous publications before; e.g^1–33^. Results obtained from detailed field/structural studies carried out on the ENS were very useful in understanding the evolution and the history of deformation of the ANS and EAO. The main objective of the present work is to shed much lights on the thrust System, flower Structures and transpressive duplexes in Zeidun-Kareim belt (ZKB) in the Central Tectonic Province of the ENS. The area is situated south of the Qift-Quseir highway asphaltic roadway on the western edge of the Central Eastern Desert. Because of it accessibility and magnificent geology, the ZKB attracted the attension of many works; e.g^34–45^. We hope that the current study will add more concepts and thoughts to the geology and tectonic setting of the ENS which would add to ANS/EAO evolution.

The geological history ties into the broader Neoproterozoic supercontinent cycle, encompassing the breakup of Rodinia, the formation and closure of the Mozambique Ocean, and the eventual assembly of Greater Gondwana^3^. During the Neoproterozoic era (900 −600 Ma), the Arabian-Nubian Shield (ANS) emerged as a complex assemblage of tectonostratigraphic terranes^46^. These terranes are characterized by well-defined suture zones, such as Duwi, Qena-Safaga, Nugrus, Kharit-Wadi Hodein, and Hamisana Shear Zones and Mubarak-Barramiya Shear Belt^47^, marked by ophiolites, which formed during the mid-Neoproterozoic period. ANS evolved in three stages: accretion, collision, and post-collision. In the accretion stage (870−670 Ma), volcanic-sedimentary sequences from island arcs and plutonic rocks were deposited on the east Gondwana continental block. A collision stage occurred between the ANS crust and the older continental margin of West Gondwana (650−640 Ma), resulting in a thickening of the crust. The post-collisional stage (630 − 550 Ma) was characterized by the extension and thinned ANS crust, as well as significant volcanic and granitic activity^48^. The main lithologies of the Egyptian Nubian Shield include metamorphosed volcanosedimentary successions, ophiolite sequences, metagabbro-diorite complexes, various granitoids formed during different stages of its tectonic evolution and the phanerozoic trachyte plugs and ring complexes; e.g^49,50,46^.

## Geologic setting

**Fig. 1:**
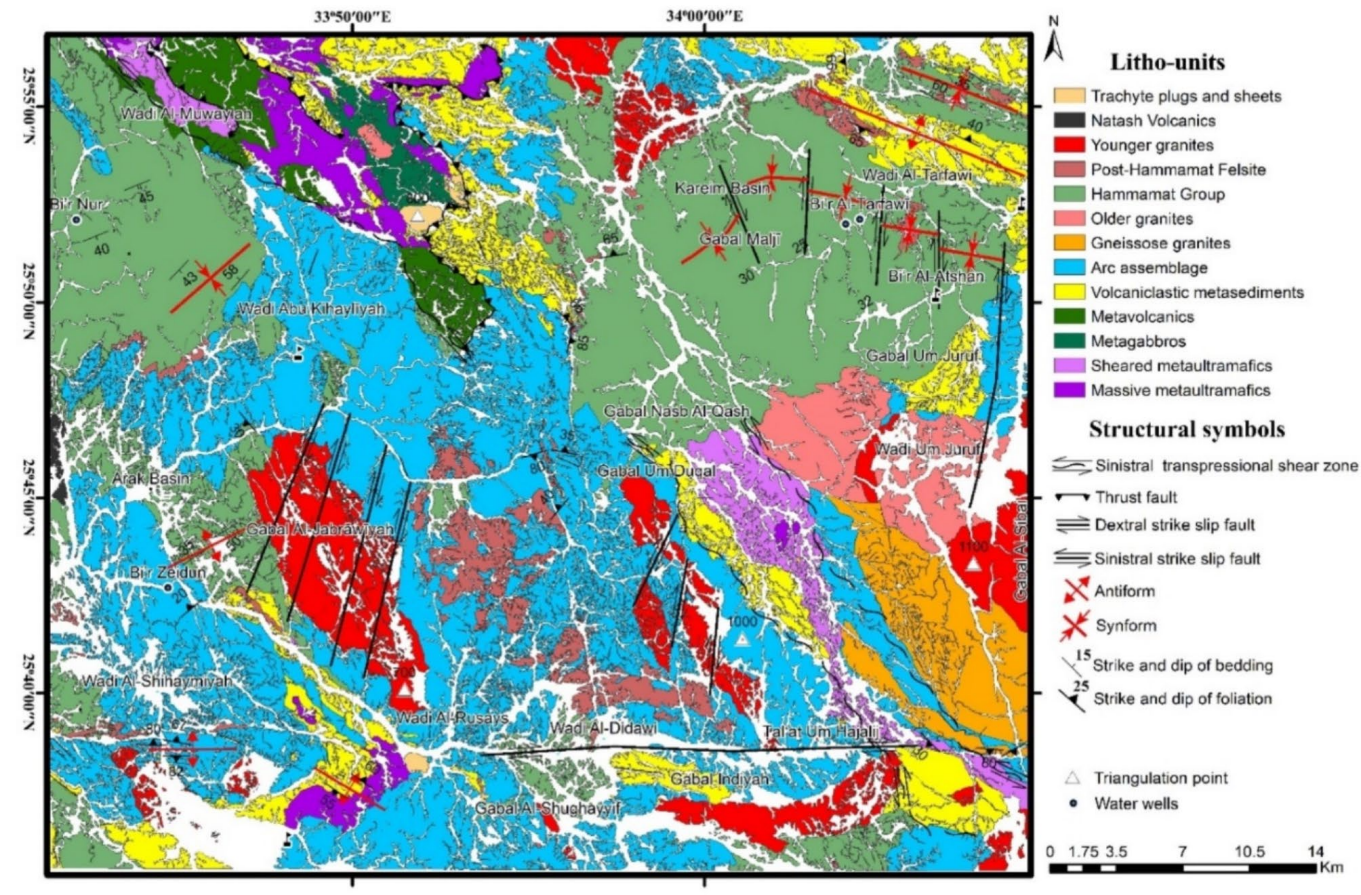
Geologic map of the Zeidun-Kareim belt (ZKB), Central Tectonic Province, Egyptian Nubian Shield. Map created by ArcGIS Desktop v 10.7.1. https://www.esri.com/en-us/arcgis/products/arcgis-desktop/overview.

**Fig. 2:**
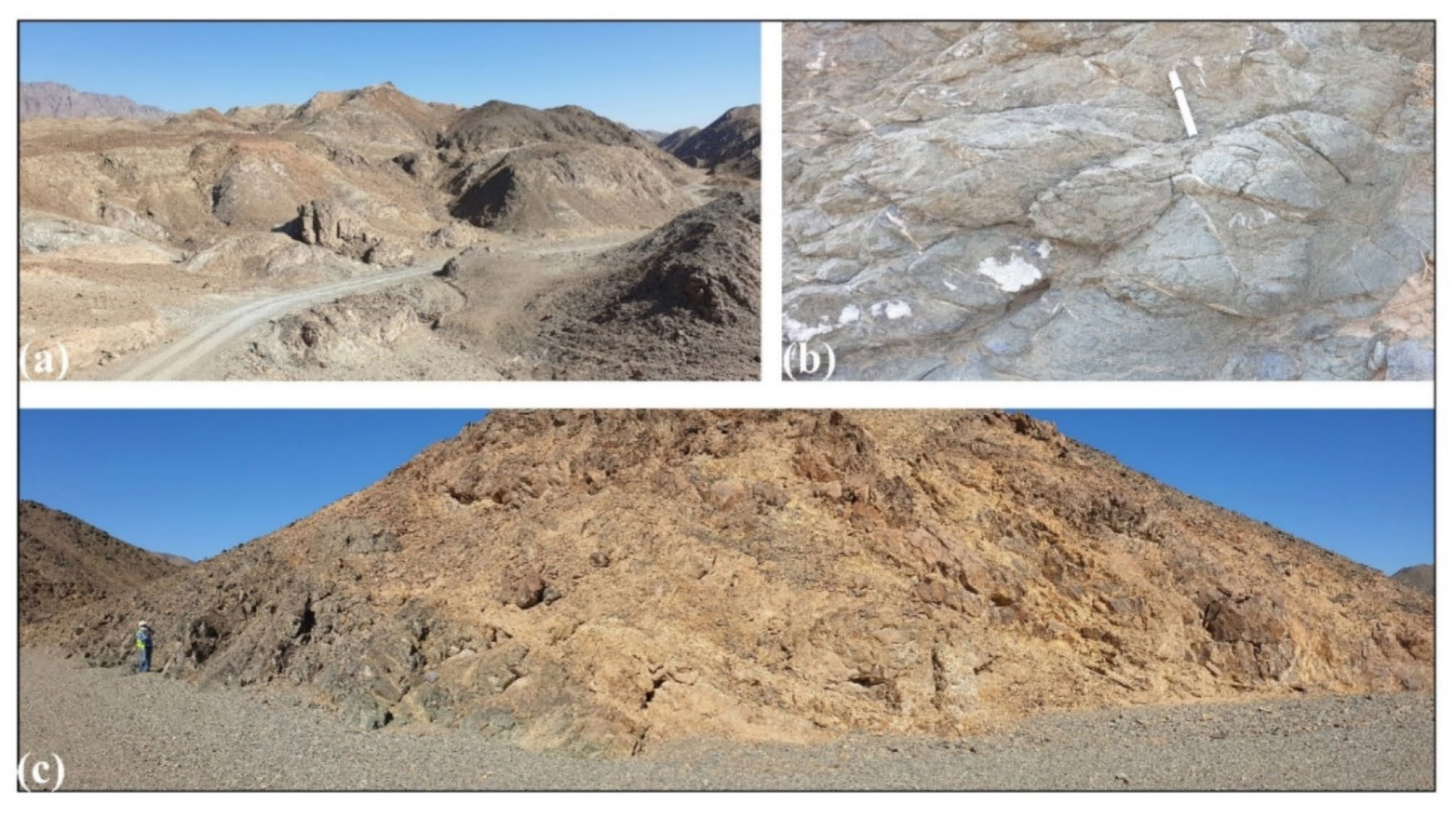
Some field relations of the exposed litho-units in the ZKB: (**a**) highly shear metaultramafics, (**b**) deformed pillows, (**c**) narrow space foliation and folding in volcaniclastic metasediments, and (**d**) thrust contact separating footwall Hammamat Sediment from gneissose granite in its hanging wall.

The area of study is covered by as series of Neoproterozoic ophiolitic litho-units representing by (Figs. 1 and 2): metaultramafics, metagabbros and metavolcanics, incorporating within a matrix of volcaniclastic metasediments. The ophiolitic rocks are characterized by a lengthy, NW-SE strip possessing moderate to high relief. They are depicted as a series of back-arc basin ophiolites made up of vast, shared ultrabasic rock associations (such talc carbonates) and metagabbros, with thrust contact situated in between. These units are directely juxtaposing arc assemblage, and the whole rock package are intruded by older and younger granites, and unconformably overlain by the Hammamat Volcanosedimentary Sequence (Hammamat Group or Hammamat Molasse Sediments). The latter lithologic group deposited in two main basins; namly Kareim and Zeidun Basins. The narrow, NW-SE elongated Hammamat small basin, which is teeteringly connected to the main basin, and the inverted heart-shaped main basin make up the Kareim basin. A portion of the basin’s current western and southern borders still have the original, unconformable contacts between the basement rocks and the Kareim basin sequence. The remainder of the basin borders are mostly tectonic, and are typically characterised by thrusted or strike-slip faulted contacts going NW or N-S. The ounger granite forms oval intrusions in some locations, such as Gabal Al-Jabrawiyah, Gabal Um Duqal and Gabal Al-Sibai, intruding the arc rock assemblage.

Post-Hammamat Felsites, Natash Volcanics and trachytic plugs and sheets do exist, particularly in the area between Wadi Zeidun and Wadi El-Qash. The felsite intrusion led to some alterations in the molasse sediments. A dyke of porphyritic microgranite with a NW trend cuts through the Arak basin. The southeast of the research area is home to the Zeidun Alkali granite intrusion, which has a NNW trend. The northern portion of the Arak basin sediments is mylonitized despite the size of this pluton. The calc-alkaline metavolcanics of Wadi Arak attain a zircon fission track age of 693 ± 40 Ma Ma^51^ and are discriminated into laminated metatuffs, meta-agglomerates, metabasalts, and metadacites, along with their associated metapyroclastics. In Wadi Kareim and Wadi El-Dabbah, the laminated metatuffs include few iron formation bands (BIF) of Algoma-type veriety. These BIF have been the subject matter of some studies before; e.g^52–55^. The metaultramafics, arc assemblage, volcanoclastic metasediments and younger granite from the North, as well as older granite from the South, encircle the Kareim basin. According to^21^, the basement rocks were put together during the arc collision stage (750–700 Ma) of the ANS. The El-Dabbah and Kareim granitoid intrusions share intrusive connections. Strong thrust faulting is also present in the basement rocks, and between the main basin and the satellite basin to its northeast lies a belt of silicic metavolcanics and heavily foliated mélange^56,57^.

## Materials and methods

A Landsat-8 level 1T (terrain corrected) image covering the present study area was processed using ENVI 5.3 (ENVI^®^ image processing and analysis software, from ITT Visual Information Solutions) and ArcGIS 10.5 (from ESRI^®^ Environmental Systems Research Institute) packages. Using the radiometric calibration tool provided in ENVI 5.3, the Landsat- 8 OLI image data were calibrated to reflectance. Processing of the Landsat- 8 OLI calibrated image using various techniques (such as: PCA, MNF, Band Ratios, and Supervised Classification) along with careful revision of previously published regional maps in the whole ENS make it possible to discriminate litho-units in the base map of the concerned area. However, obtained results from remotely sensed data will be dealt with in another publication. In the base map, several cross- and along-strike geologic traverses were determined for ground truth. During the filed program, the materials were collected from vaious litho-units outcropping in the ZKB. The area has been examined through a great number of traverses. In each traverse, field observations, overprinting relations and structural measurements of orientational data were collected from specific observation points (stations). Attitudes of the orientations data and oriented samples were measured using the Brunton Compass. Thin sections prepared from both representative- and oriented- samples were carefully investigated under the Nikon E600 POL Reflected & Transmitted Polarizing Light Microscope. FaultKin 8.1 software^58^ is used to analyze fault-slip data from the field and export data as a stereonet. Macromedia FreeHand MX 11 was utilized to trace the field photos and create sketches. Adobe Photoshop CC 2018 (32 Bit) was used to combine the field photos, sketches and stereonet, and enhancing them together in their final form. The outlines of deformed pebbles were traced, and digital (BMP) files were created for each image captured from outcrops and thin sections using Macromedia FreeHand MX 11. Digital image processing was conducted with the Semi-Automatic Parameter Extraction Program (SAPE)^59^ to extract strain parameters from grain boundary traces of deformed pebbles. These parameters include the long axis (a), short axis (b), axial ratios (Rf) typically falling between 40 and 105, and orientation data (φ) extracted along the line of maximum stretching from the BMP image by SAPE. Finite strain analysis in deformed rocks was determined using the Rf/φ method to analyze aggregates^60,61^. This technique relies on calculating the ratio of the long axis to the short axis (Rf) and the orientation (φ) to construct the Rf/φ diagram. By comparing these values with standard strain curves from previous studies^62^ and considering the elliptical or sub-elliptical shapes of deformed aggregates^63–66^, recent computer software advancements have made it possible to accurately calculate finite strain using the Rf/φ method. Authors like^62,67^ have contributed to enhancing this method over the years. To generate Rf/φ diagrams for finite strain analysis, the method outlined by^68^ involves utilizing a spreadsheet known as the CSS. This spreadsheet is comprised of four worksheets, where users input the strain parameters representing the long and short axes (a), (b), (φ), and (Rf) on the initial sheet labeled “Enterdata.” The process entails extracting only the Rf/φ values from the SAPE program and saving the data in a file format with the extension (RFP). Subsequently, these parameters are imported into the MRL (Mean Radial Length) software developed by^69^ to facilitate finite strain calculations.

##  Results

### Directions of thrusting

**Fig. 3:**
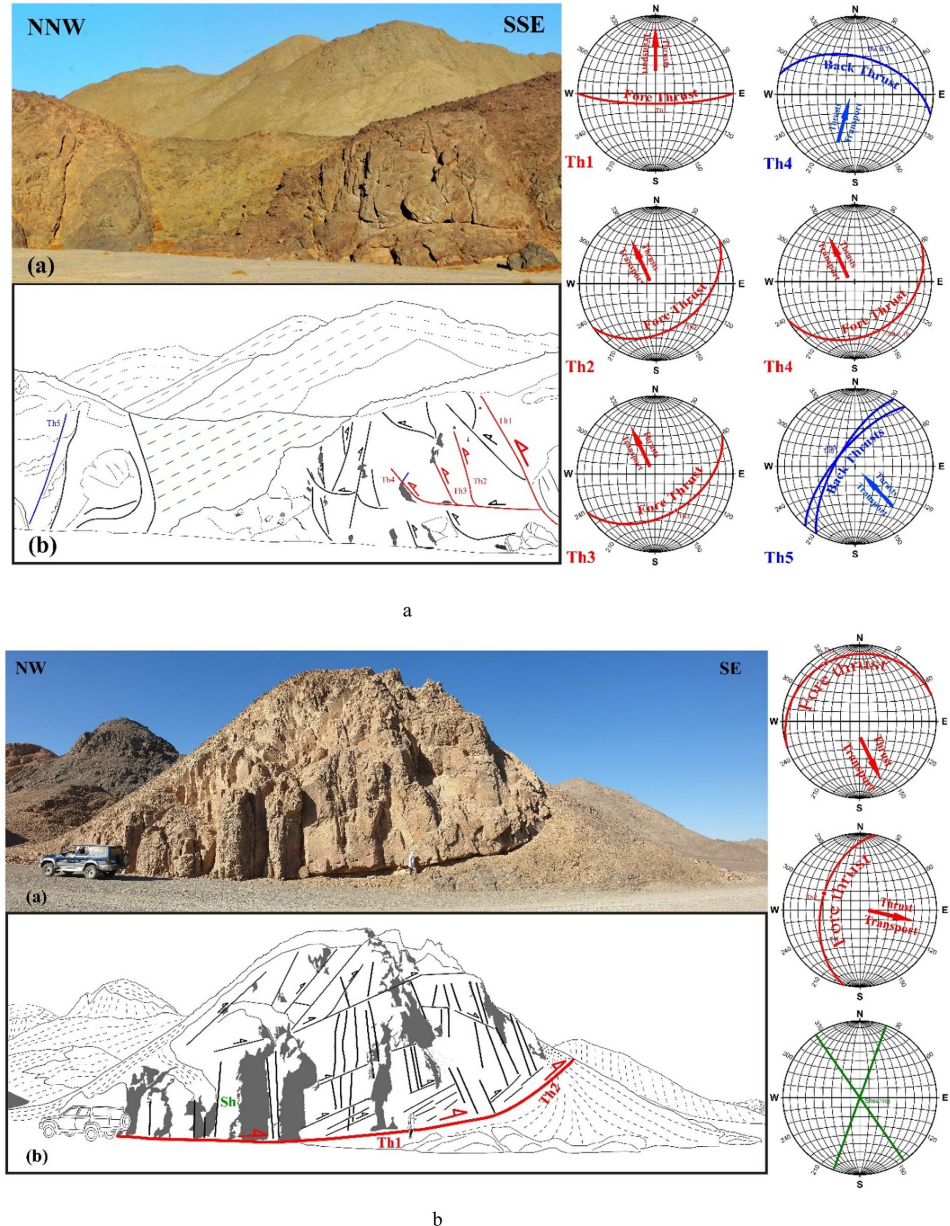

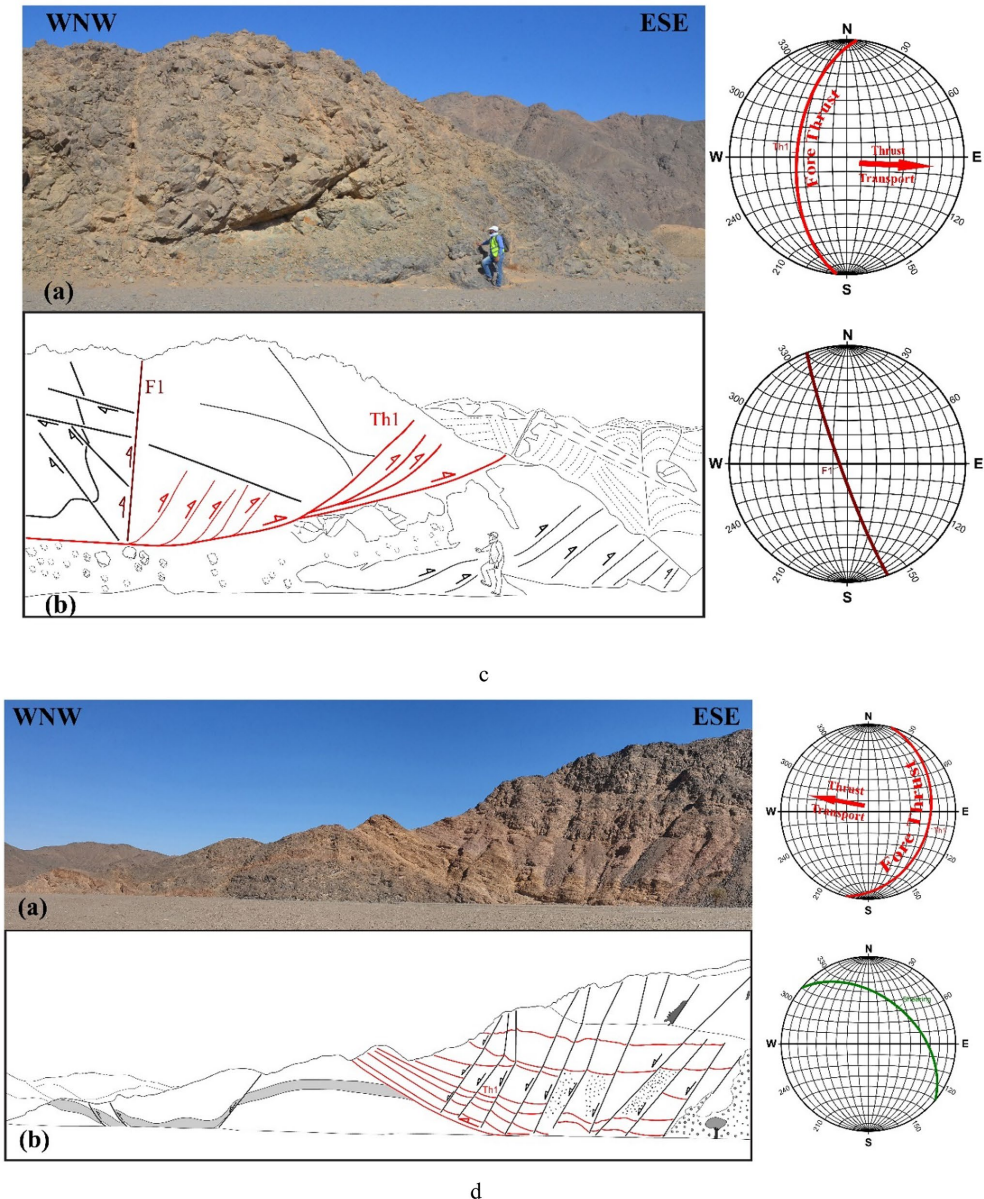

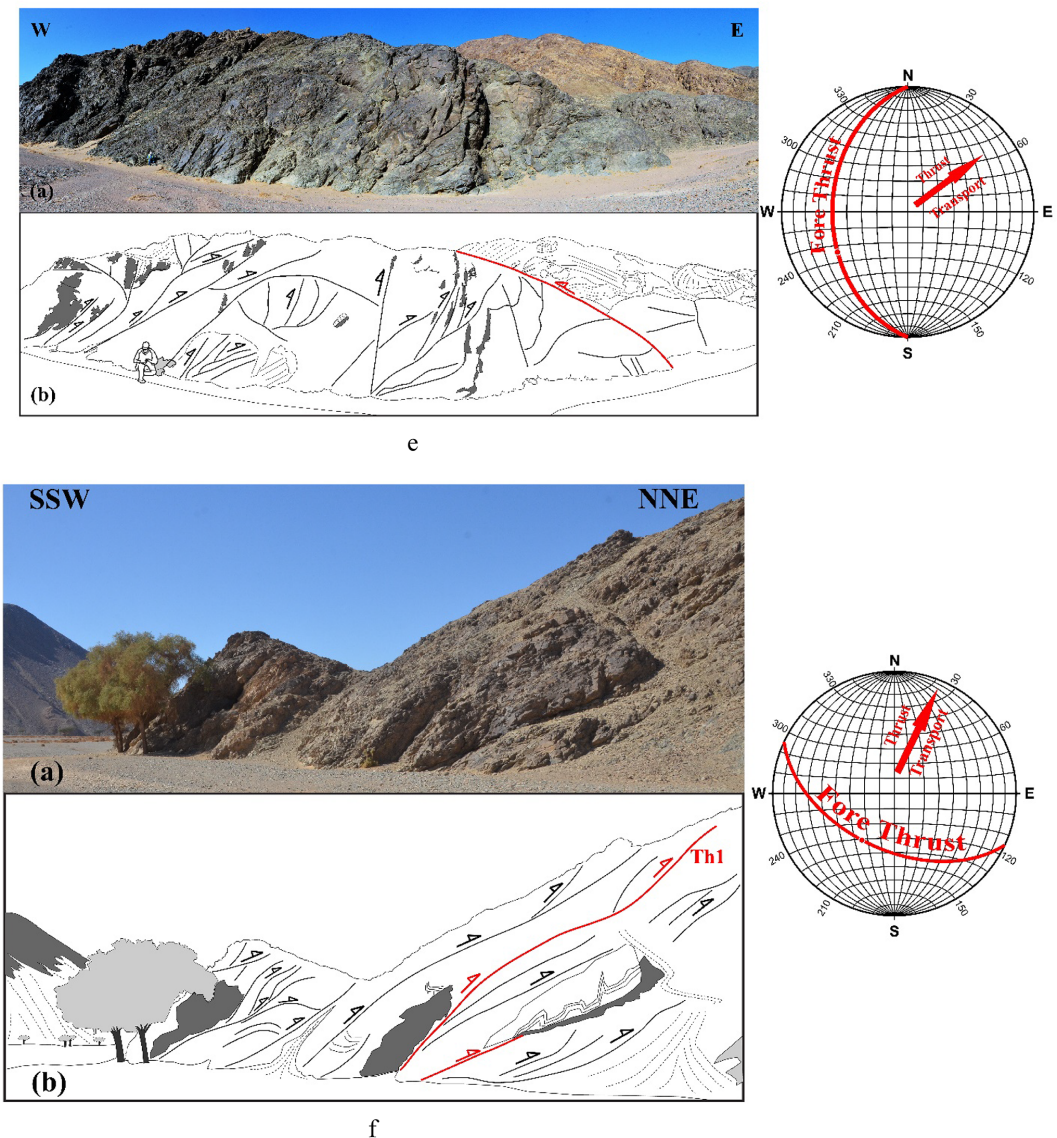

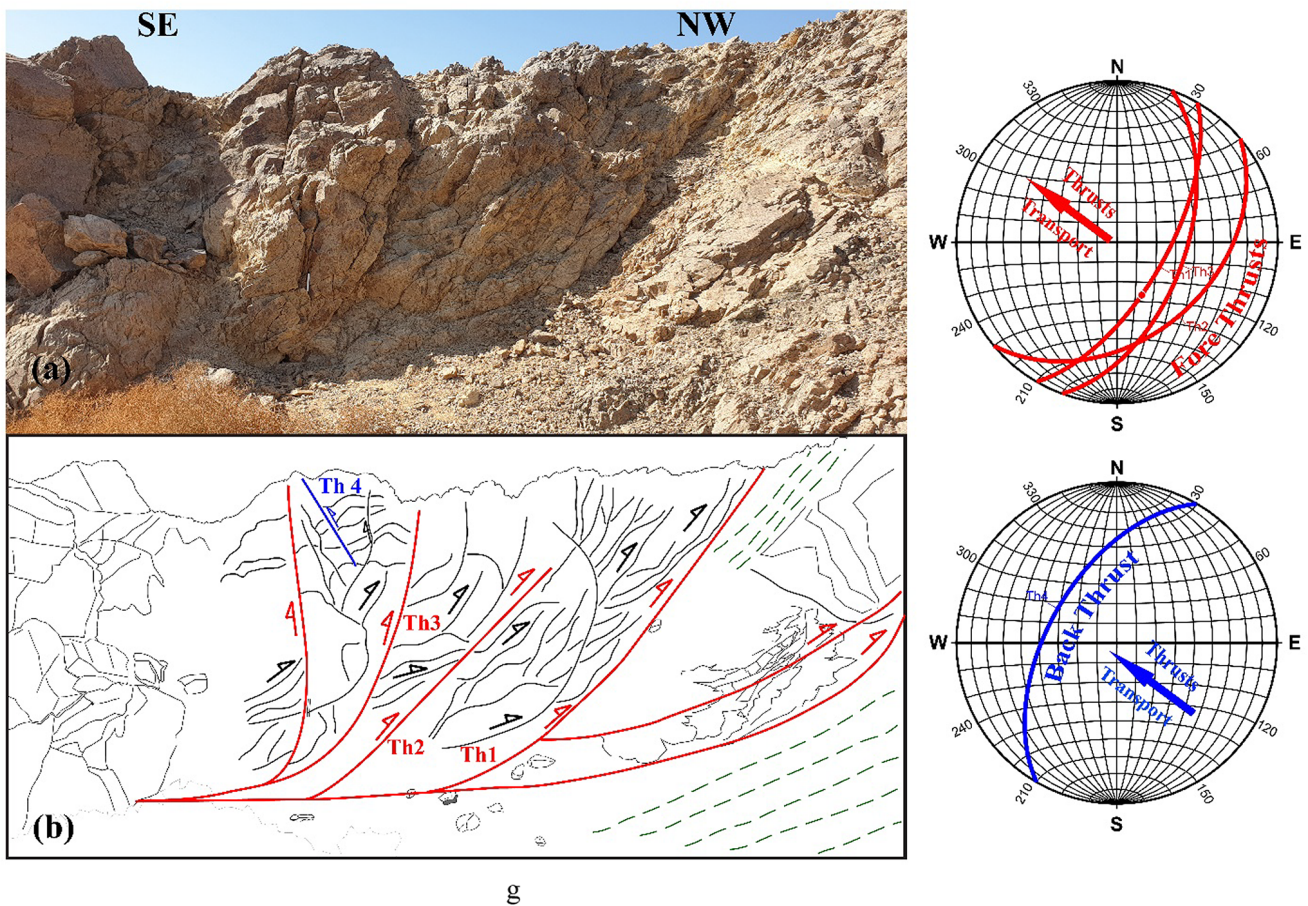
Field photographs showing well developed thrusting at an outcrop-scale, with sketches for clarification. Attitudes of propagated in-sequence foreland propagated thrusting (and if existed back thrusting) and directions of transport are shown in the stereonets; from 3a to 3 g: SSE-, SE-, ESE-, E-, NNE- and NW-vergent thrusting, respectively.

**Fig. 4:**
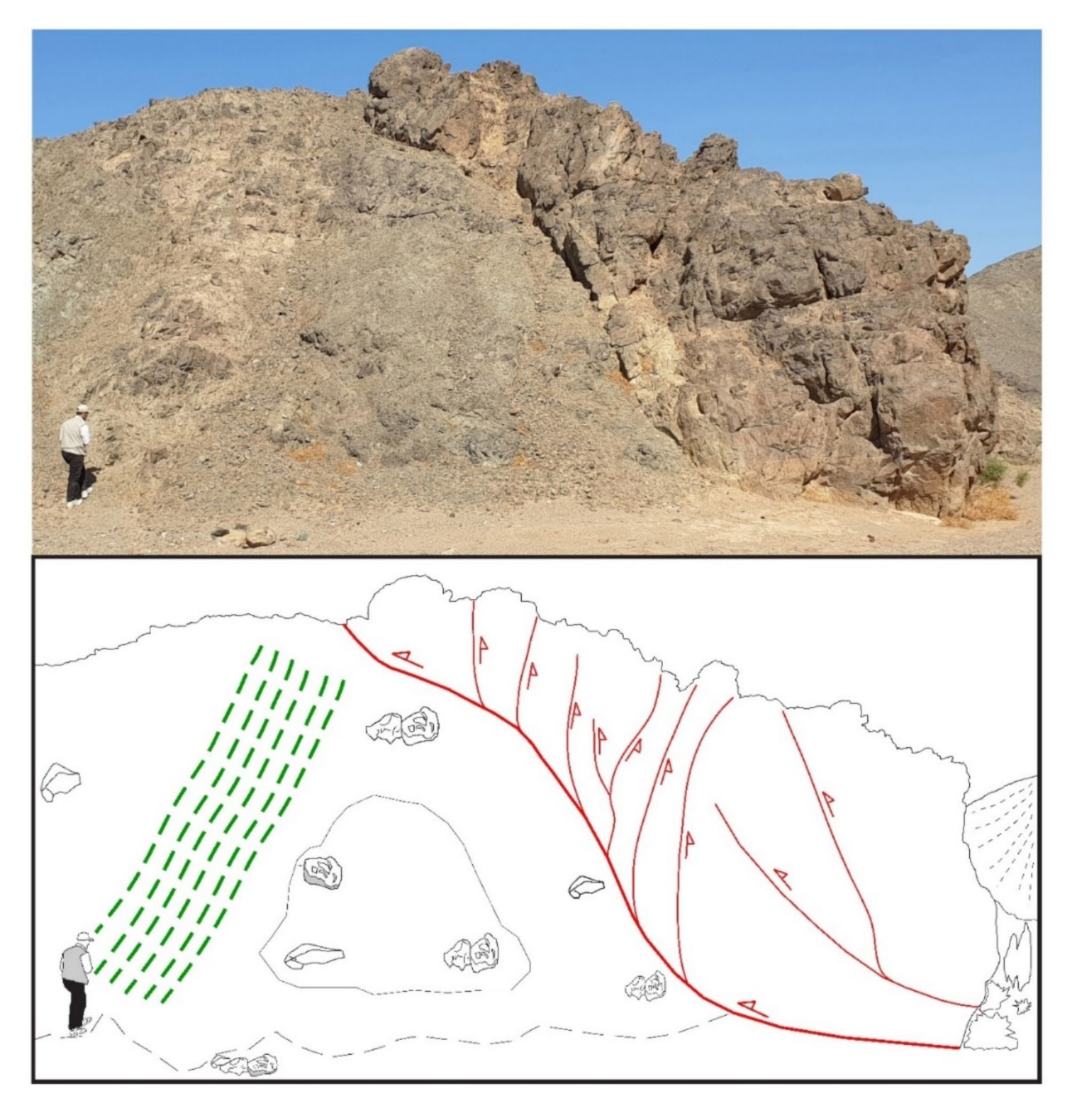
Flat-ramp-flat geometry with subhorizontal detachment (décollement) surface and steeply dipping ramp. Notice how stacked horses created.

**Fig. 5:**
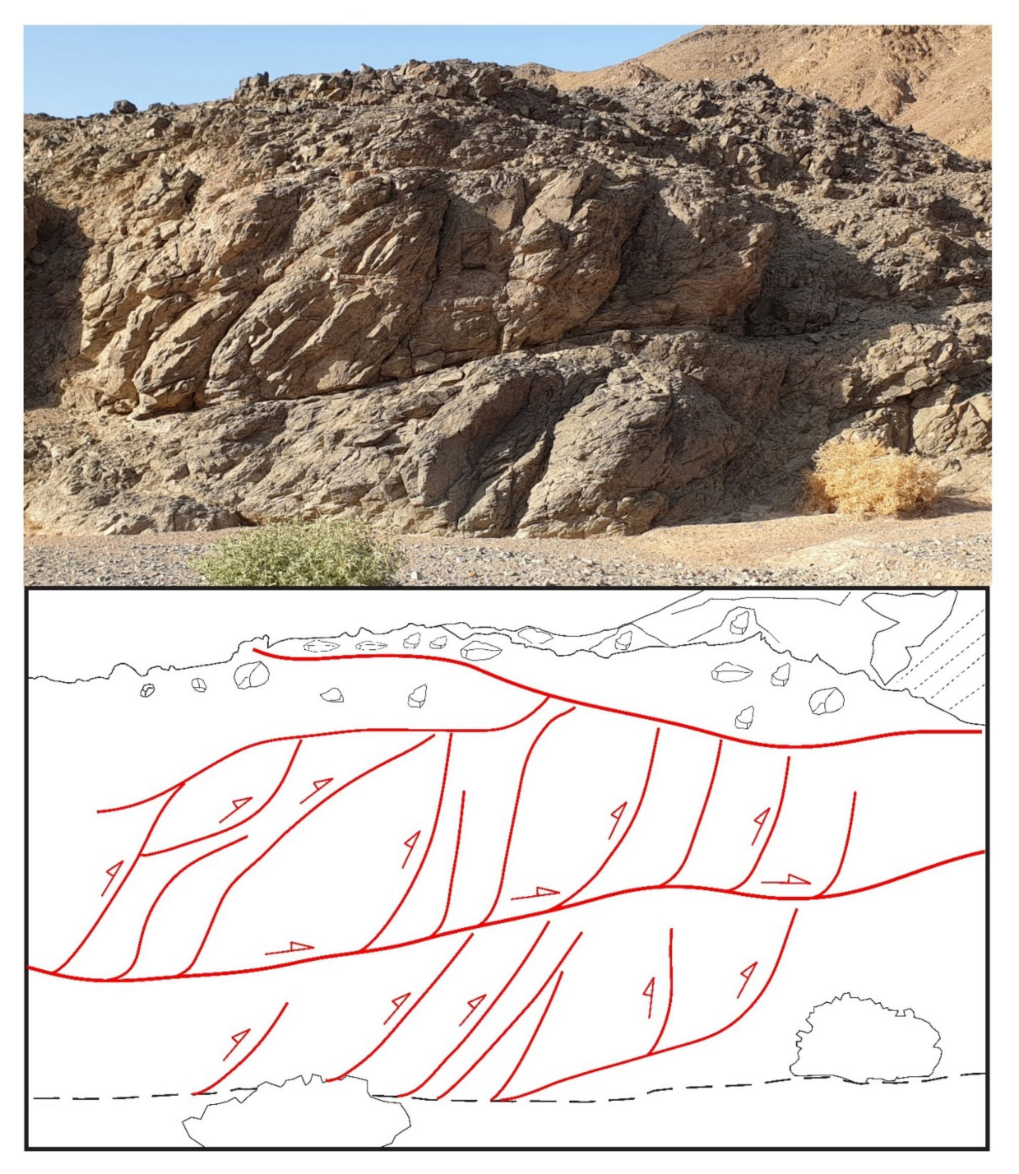
Wonderful thrust duplex comprising nearly flat décollement-surface (sole thrust) and roof surface and in between imbricated horses marked by inclined ramps.

In ZKB, field investigation and collected orientational data indicate that thrusting shows different directions and vergences. Top-to- SE-, E-, ESE-, NE-, NNE-, NW- and WNW- thrusts are dominant (Fig. 3a–g). Although these thrusting directions seem to be affiliated to five thrusting phases, in the present study because of the absence of overprinting relations, we proposed only two main thrusting phases. The first phase of thrusting was responsible for the formation of the SE-, E- and ESE-vergnet thrusts, besides the NE- and NNE-vergent thrusts. During the second phase of thrusting, NW- (to WNW-)-vergent thrusts are formed. Separation of the two thrusting phases is based on the measured stretched lineations. Thrusts of both phases are most probably formed under semi-ductile semi-brittle conditions. They are observed at a wide variety of scales ranging from microscopic-scale up to outcrop-scale. The dip angles of thrust planes are frequently relatively steeper in the down-dip direction compared to the up-dip direction in which the angles are mild or gentle. Occasionally thrusts are detected by the vergence of the accompanied thrust-related asymmetrical folds which are formed because of the propagation of thrusting; thrust-first kinematics. The thrusts generate sequentially in “in-sequence” or “piggyback” manner according to the footwall-nucleating-footwall-vergent rule, where younger thrusts tend to form in the footwall of the earlier thrusts. They often show listric or flat-ramp-flat geometry with subhorizontal detachment (décollement) surfaces and moderately to steeply dipping ramps (Figs. 4 and 5). Single and multiple detachment or décollement surfaces are frequently observed. The single detachment surface often separate remarkably deformed litho-unit in its hanging wall from less deformed another unit in its footwall. Such surface is often characterized by the presence of a narrow zone of mylonite. Ongoing of in-sequence thrusting led to rotation of the earlier formed thrust to inactive planes with high dips to accommodate the subsequent deformation as described in many fold-thrust belts; e.g^70–91^. In some outcrops, “out-of-sequence” (or “break-back” ) thrusting is observed, and in such case these thrusts show asymmetric pattern and initiate in the hanging-walls of the earlier formed thrusts. These thrusts do not get systematically younger towards the foreland^92^ and tend to form in the hanging walls of earlier formed in-sequence thrusts.

### Flower structures, transpressive duplexes and related structures

Propagation or stacking of the top-to- SE-, E-, ESE-, NE-, NNE-, NW- and WNW verging thrusting frequently occurred under the greenschist to the lower amphibolite conditions in the present study area. The propagation rate is a consequence of the shortening component. This involves variable degrees of internal deformation and formation of a great number of thrust-related structures, thrust duplex and thrust-related folding within individual litho-units, besides transposition foliation. Of course, appearance of these structures varies from an outcrop to another, depending on many factors like the ductility of the rock unit, the deformation state and the stage of development.

### Flower structures

**Fig. 6:**
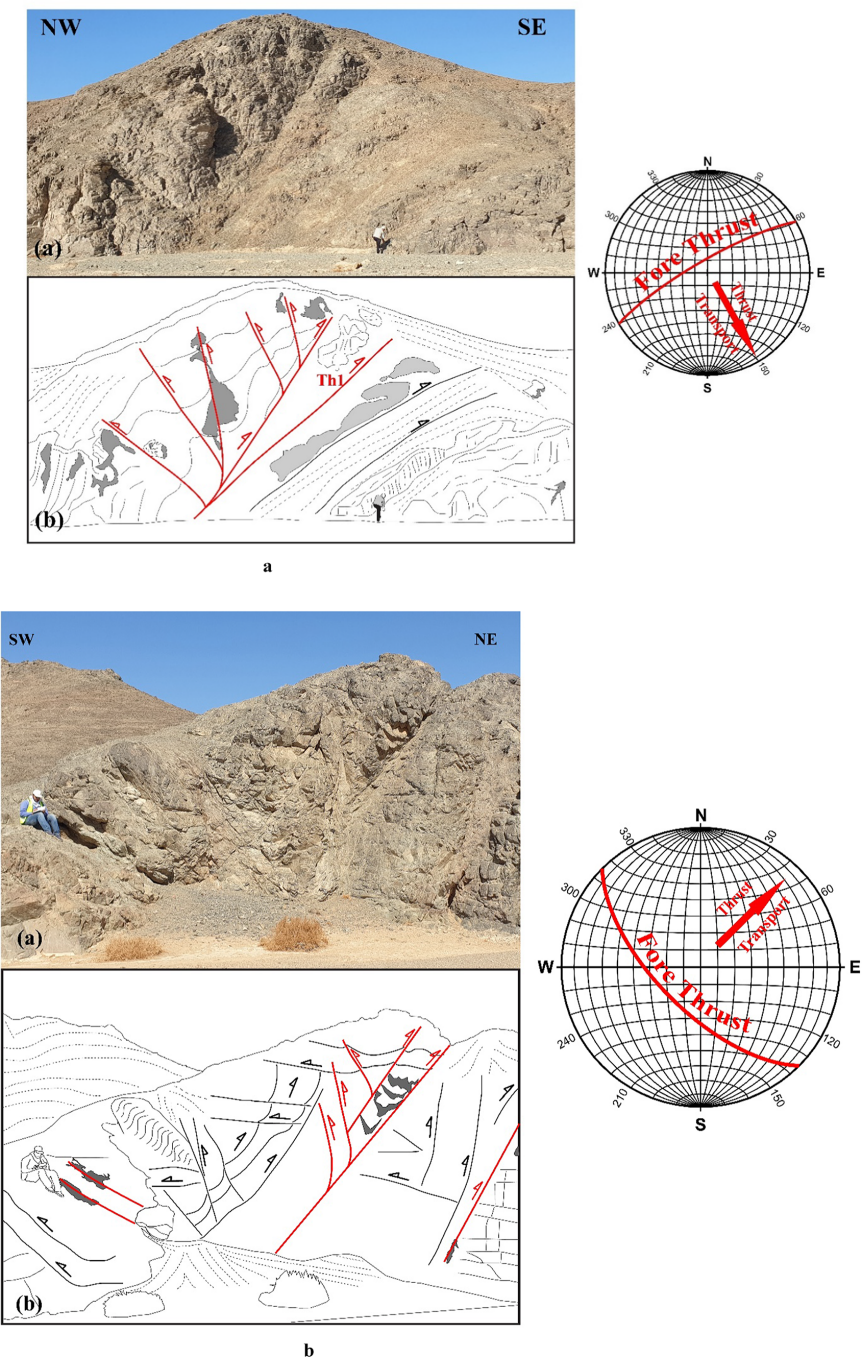
(**a**, **b**) Positive flower structures formed due to the combination of thrusting and transcurrent shearing in the ZKB.

In addition to the abovementioned structures, well-developed and magnificent positive flower structures are observed in the ZKB (Fig. 6a, b). These structures are most significant in deducting the transcurrent shear zones or wrench fault zones in regional-scale studies; e.g^93–95^. They are characterized by their own architecture and internal deformation, and often encompassing shallow antiforms and upward dispersal strands of thrust faults. The widespread occurrence of the positive flower structures that were formed due to the combination of thrusting and transcurrent shearing indicates that thrusting in the ZKB is not a simple-pure thrust system but rather a transpressional regime. The term transpression coined by^96^ and mathematically modelled by^97^ who were able to define its boundary conditions. In (1997)^98^, extended the transpressional boundary conditions to allow for lateral extrusion tectonics. However, the prevailing of flower structures makes the ZKB as an ideal belt throughout the ENS or even the entire ANS to deduct and interpret the intimate relation of thrusting to shearing. In other words, the ZKB flower structures offer good insights into how thrust system and shear zones evolve with time. Such tectonic regime point out that thrusting and shearing are geometrically- and kinematically-related and interpreted to be linked. But, whether thrusting took place first or shearing or even both were simultaneous, this is equivocal, questionable and can’t be deciphered, particularly if we take into consideration that contraction component is clearer than strike-slip component, and vice versa, at the outcrop-scale.

### Transpressive duplexes

The contraction or thrusting component, which is in most cases oblique-slip in the present study area, can obviously attested by investigating the subvertical exposures with noticeable thrusting planes, thrust duplexes and asymmetric verging folding with long upper limbs and short overturned lower limbs, as well as by inspecting the stretching lineations along thrust ramps. Sometimes, the contraction or the propagation of thrusting may have led to amalgamation of earlier formed thrusts and thrust-related features, and eventually formation of new through-going fabrics.

In the ZKB, marvelous thrust duplexes consisting of detached slices and stacked horses (imbricated lenses or imbricates) are observed (Fig. 5, see also Fig. 3a). Each duplex comprises a sequence of ramps bifurcated from nearly flat décollement-surface or sole thrust (floor thrust) marked in some cases by a very thin veneer or narrow zone of mylonite. The ramps megre and coalesce upward with an upper roof thrust. While the angle between a ramp and the sole thrust is nearly tangential, it is high with the roof thrust. Also, the dip anlge of the ramps themselves become steep in the hinterland and progressively increase towards the foreland; towards the propagation direction. Because ramps and horses dip toward the hinterland and opposite to the foreland, the created duplexes are known as hinterland-dipping duplexes, and these duplexes are prominent in most fold-thrust belts. A duplex begins to initiate because the thrust is forced up to ramp up to a higher slide horizon due to the presence of a sticking point. Ongoing of the thrust propagation led to gliding along the newly formed ramp and further stacking may lead to horses or even antiformal stacks. Moving of rock slice sup the ramps may lead to bending and folding, and consequently the formation of fault-progagation folds. The shape of these folds influenced by some parameters such as ramp dip, displacement rate along the décollement surface and the propagation to slip ratio. Of important to denote here that the majority of the encountered duplexes in ZKB are active-roof duplexes that formed under low shortening rates in which roof components move forward (as horses). In this type of duplexes, flat-on-flat geometry is attributed to the long-distance translation of the borse slices or blocks. Occasionally, passive-roof duplexes that formed under high shortening rates and exhibiting infrequent flat-on-flat geometries do exist associated with back-thrusting, and in such a case the roof components move opposed to the horses. The back-thrusting led to moving the hanging wall in the direction of the hinterland or opposed to the main transport direction^80^. At the outcrop-scale we can discriminate between primary- and secondary-back thrusting. The primary back-thrusting often propagates form the basal decollement and sometimes traverse the fore-thrust ramps, whereas the secondary back-thrusting frequently initiate near the end of the upslope of the fore-thrusting and die out rapidly. According to^99^ the relative strength of the décollements and surrounding rocks affects the development of active- or passive-roof duplexes (triangle zones), and the passive-roof duplex with spaced anticlines forms in response to a strong de´collement or to rapid shortening. The shortening rate and strength of the detachment surface or décollement play an important role in the growth and timing of formation, as well as the propagation of structures within thrust duplexes^99^. Besides the above mentioned model, two other kinematic models have been proposed to discuss how this type of duplexes initiated; (1) the prograding monocline model which requires a great amount of underthrusting^100^ and the spaced-ramp anticline model in which the underthrusting happens locally overhead each horse slab or sheet^101^.

### Shear Zone-related folding, S-C foliation and other kinematic indicators

**Fig. 7:**
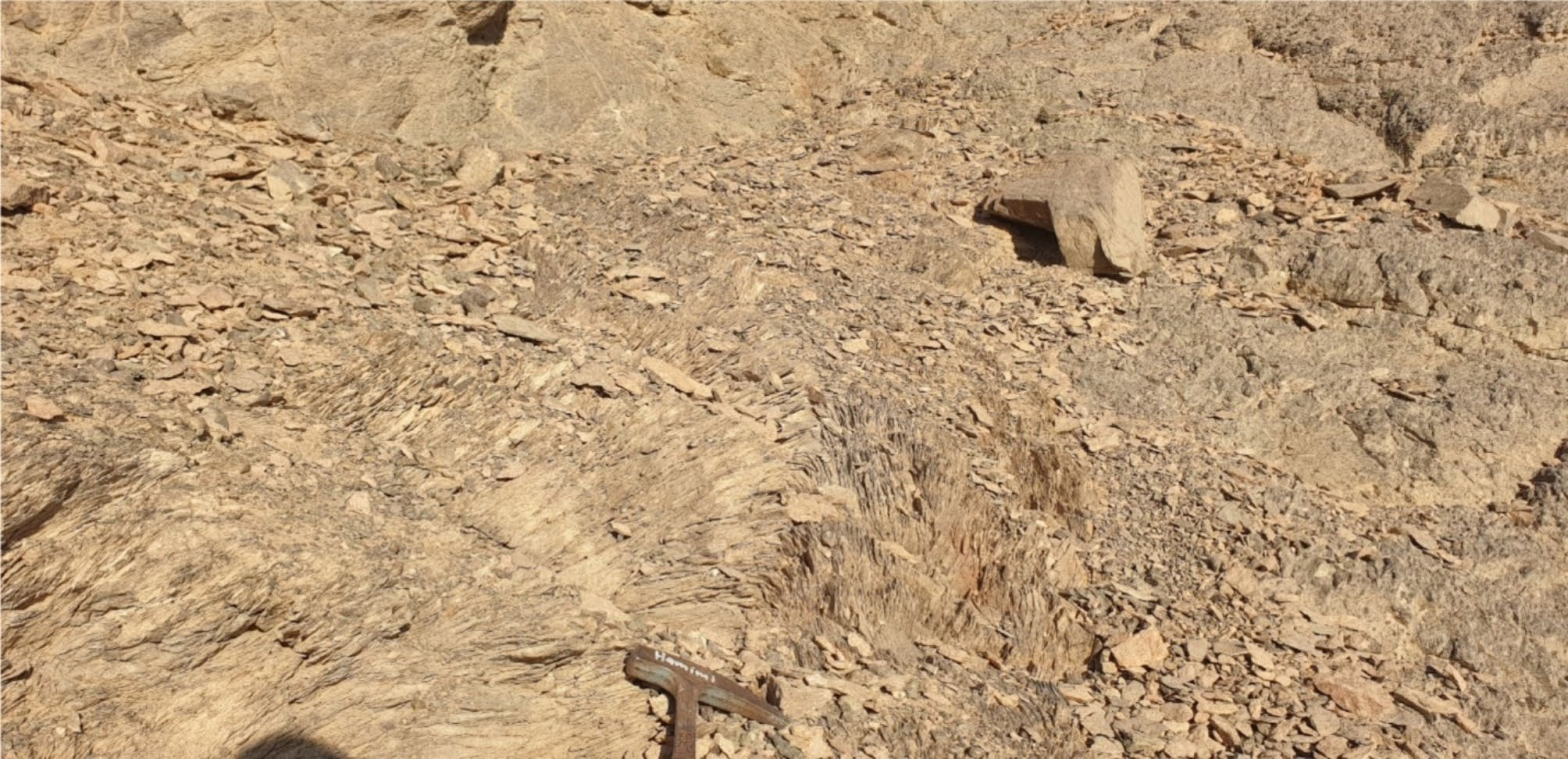
Shear zone-related folding formed in a progressive stage of shearing in the ZKB.

**Fig. 8:**
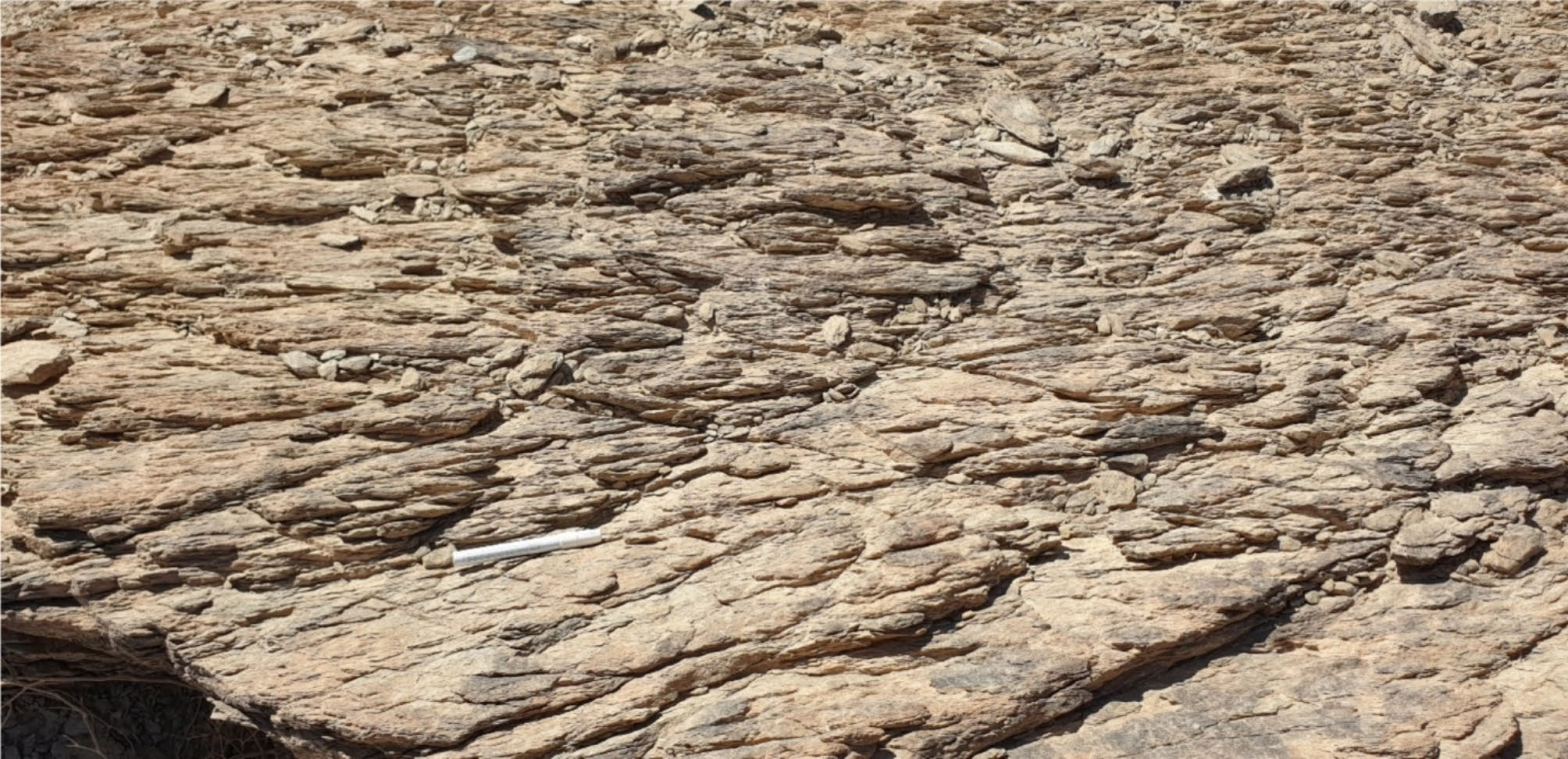
S-C foliation in volcaniclastic metasediments encountered in the eastern part of the study area, reflecting sinistral sense of shearing.

At subhorizontal planes, structural features and numerous kinematic indicators, such as shear zone-related folding (Fig. 7), S-C foliation (Fig. 8), steep dipping of frontal thrust ramps, deflected bands and asymmetric porphyroclasts and mineral fish at microscopic-scale along with other indicators with monoclinic symmetry, as well as elongated low ridges (forebergs) reveal that all of which result from strike-slip movement or transcurrent shearing. Such movement may lead to the formation of shear zone-related folding and occasionally restraining bends in few exposures.

### Quantitative strain analysis along thrust planes

**Fig. 9:**
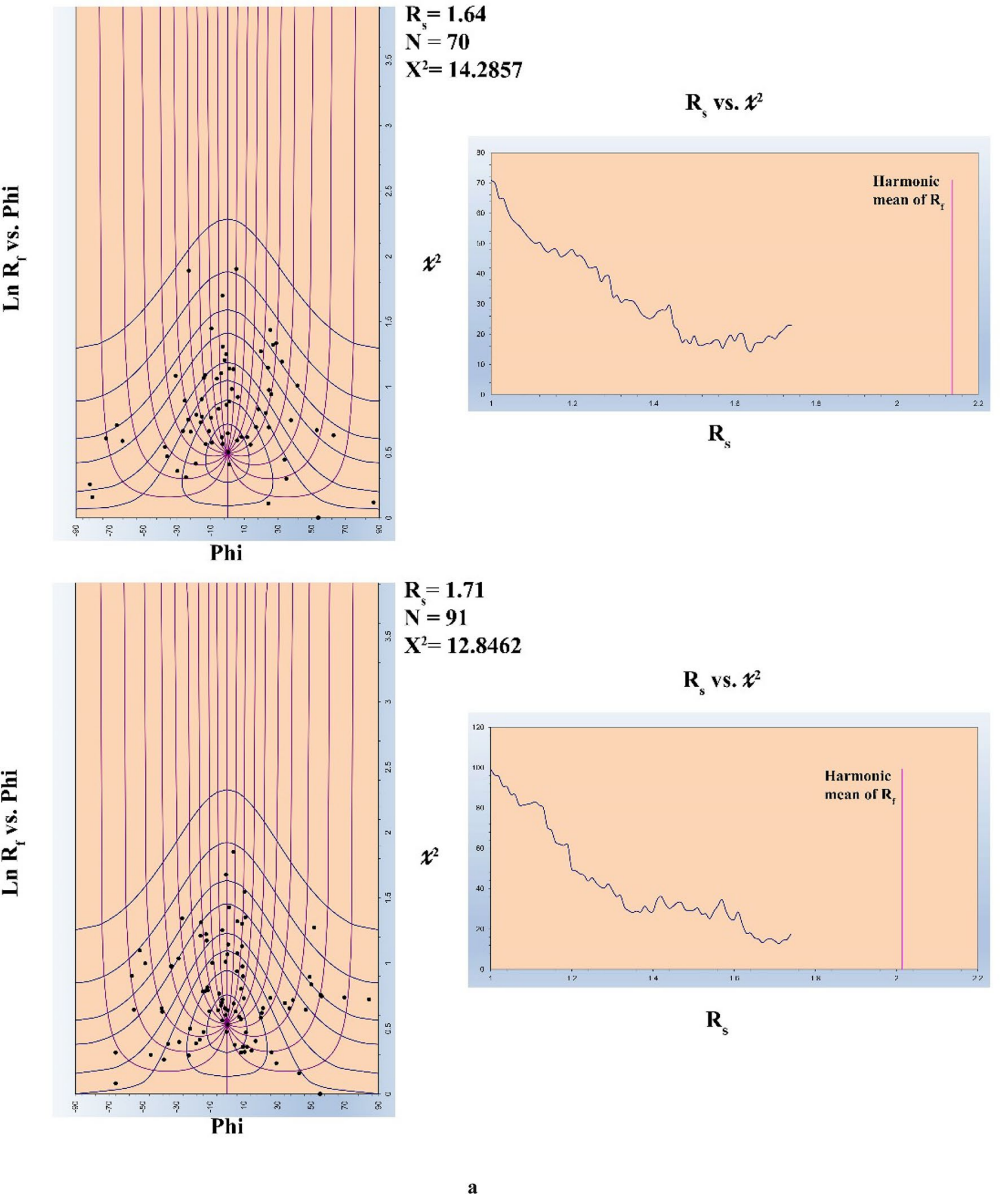

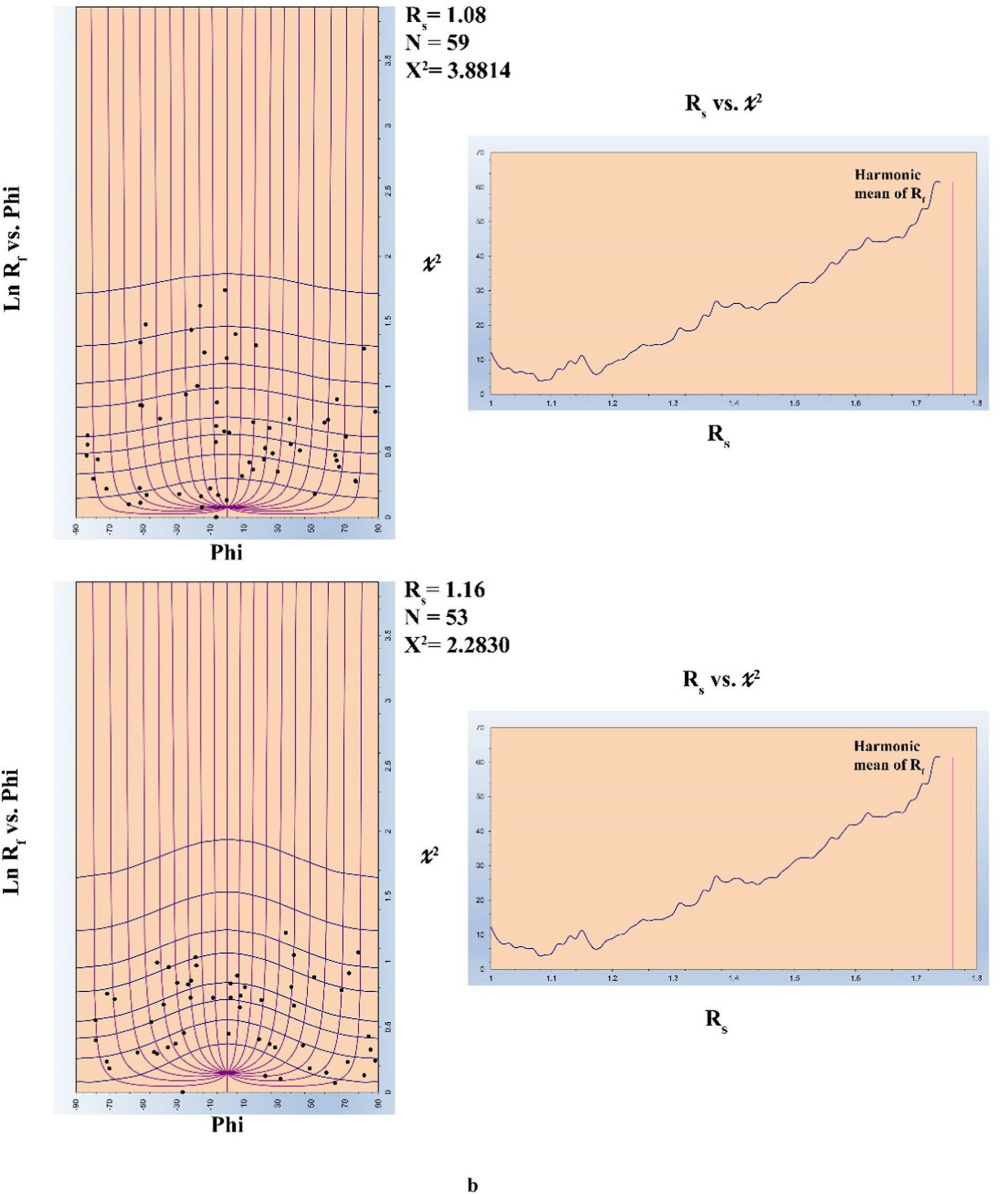

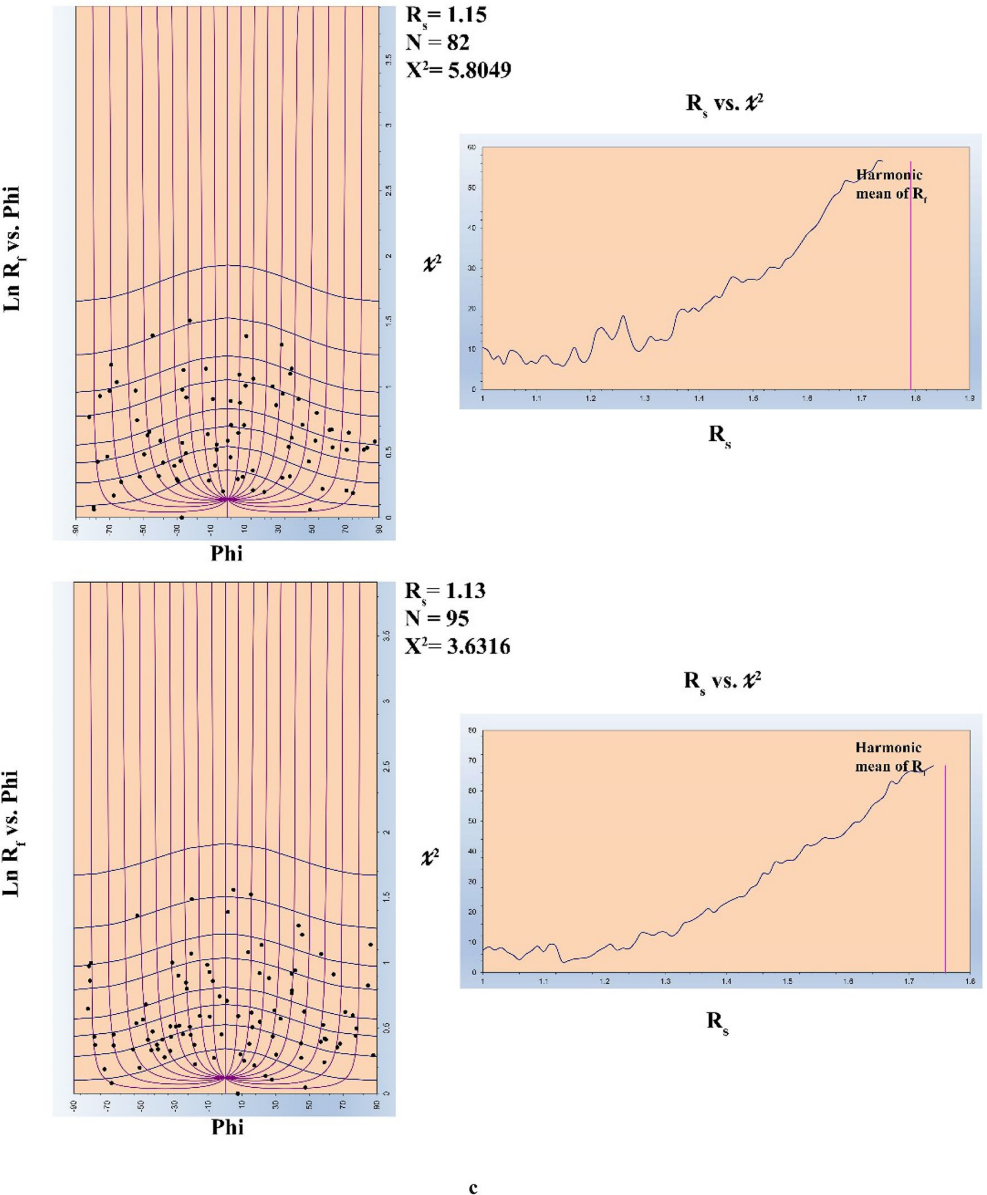
Strain ratios along one of the main thrust in the ZKB from (**a**–**c**).

Quantitative strain analysis carried out along some listric thrusts that splay out from mild décollement surface to steeply dipping ramp shows a little bit increase in strain ratio (Rs), but this can’t be considered as statistically significant differences of Rs with the variation of dip angle (Fig. 9).

## Discussion

The main objective of the present study was to investigate the thrust system in ZKB, one of the key belts not only in the ENS but also in the entire ANS. During field work, we recognized that thrusts have various directions and associated with a great number of thrust-related structures, such as thrust-related folding and thrust duplexing. At the same time, as the ZKB lies in the Central Transpressional Tectonic Province of the ENS^102^, it was expected that thrusting should be combined with Najd-related Transcurrent Shearing, which means that both thrusing and shearing are intimately-related. For this reason, our fieldwork program began by asking some enigmatic questions and aiming to find logical interpretations of the geologic structures prevailing in the ZKB. Taking into consideration that both types of structures are prominent features in the area, the first question came to our mind, what is the geometric relation of thrusting to folding? Next, we asked what is the relation of shearing to thrusting, and whether or not shearing and thrusting are geometrically- and kinematically-related? Another question was do we have reasonable evidence supporting transpressional structuring or transpressional regime in ZKB? Finally, we asked how all stuctures were formed in order to assess the evolution of the ZKB?

### Geometric relationship between thrusting and folding

Careful inspection of both thrusting and folding encountered throught the whole ZKB reveals that both types of structures exist next to each other at the outcrop-scale and even at the handspecimen- and microscopic scales. The overtuned style of folding (with long upper lime and short lower overturned limb) which is frequently bounded by lower detached suface (décollement) and upper roof thrusting or by thrust ramps in the subvertical sections points out that fold geometry is related to thrust geometry. Several line of evidence support thrust-first kinematics which means that thrusting preceeds folding. Among these evidence is the occurrence of folds between thrusts and the active bedding planes with remarkable slip lineations of folded layers. This means thrusting preceeds folding and we have thrust-first kinematics. The dip angles of thrust planes, thickness of the imbricated and stacked thrust sheets and slip rate are factors controlling the geometry of folding. Addressing the foregoing questionable points highlighting and confirming the novelty and originality of our work.

###  Interaction between shearing and thrusting

**Fig. 10:**
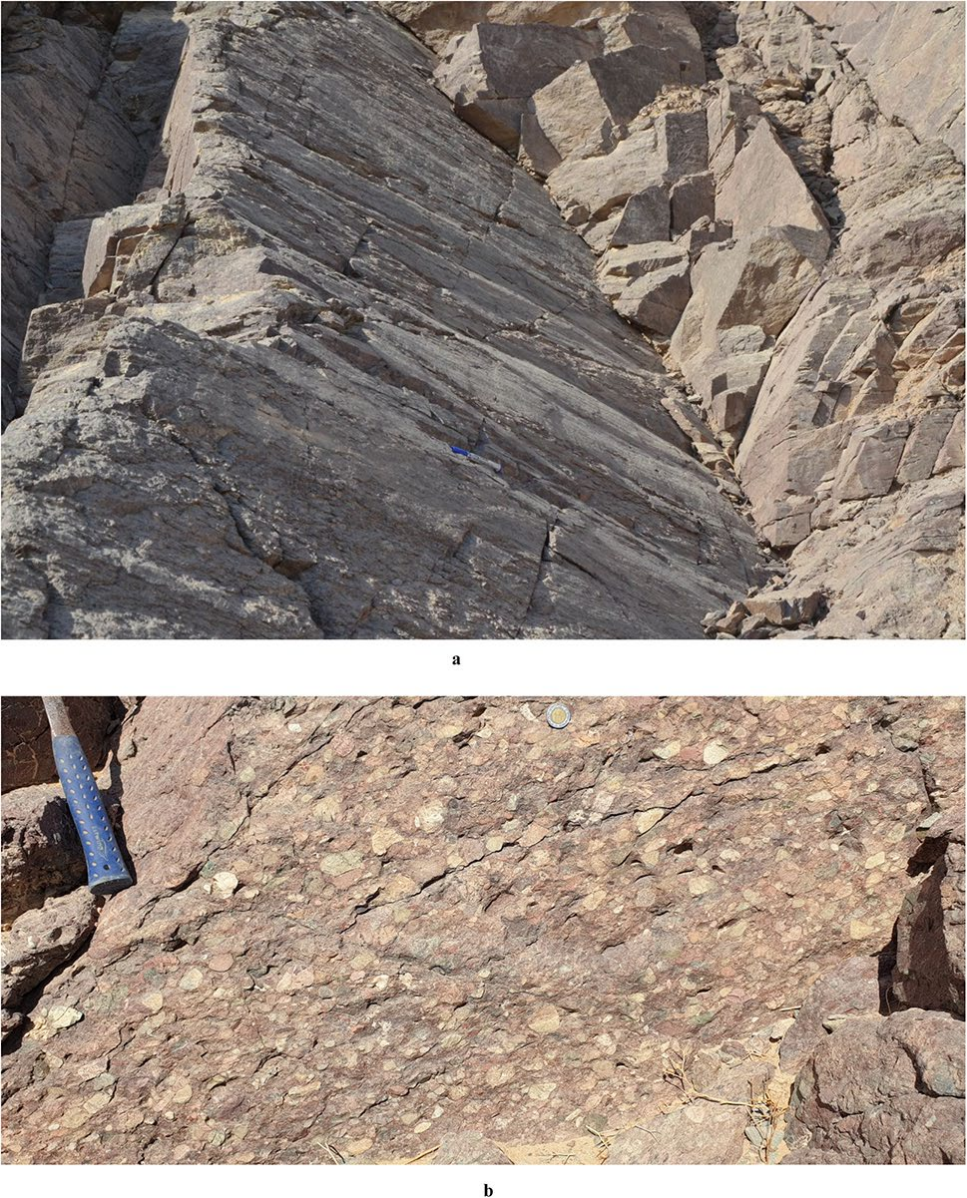
(**a**) Oblique-lineation encountered along transpressive plane, near Bir Kareim; (**b**) Moderately plunging stretched Hammamat pebbles recorded along a shear zone.

**Fig. 11:**
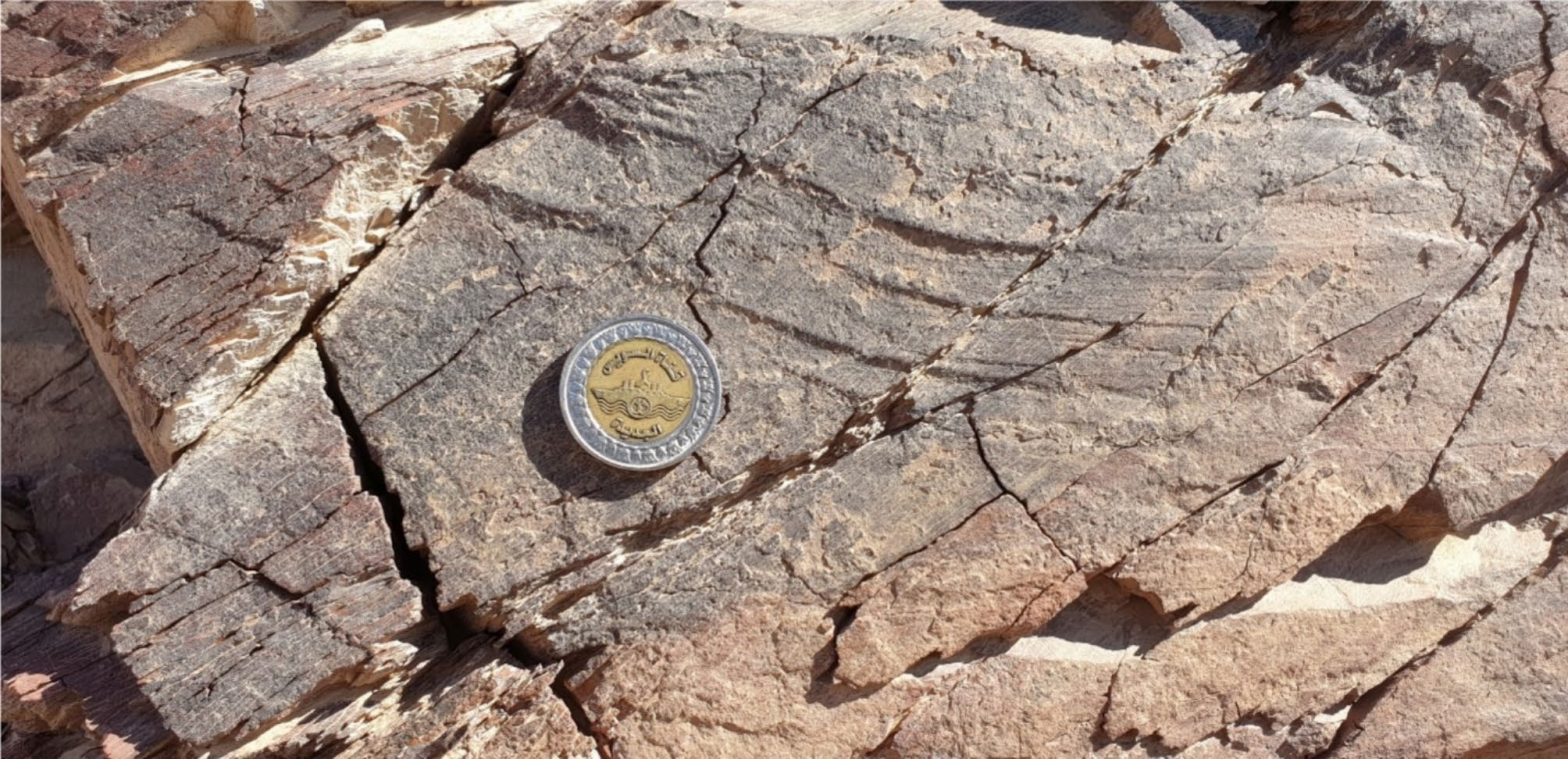
Curved stretching lineation in minor shear zone traversing weak thrust planes.

Shear zones are high strain zones formed under ductile-, brittle-, semi ductile-semi brittle or semi brittle-semi ductile tectonic regimes. Ideal shear zones as defined by^103,104^ have straight and parallel undeformed walls bordering remarkably deformed zones, exhibiting identical shearing and constant strain. Such ideal shear zones are regarded to be formed by simple shear and/or compaction-dilatation. The narrow tectonites (including both the mylonite zones and the foliated cataclasites) that are recorded along most shear zones formed under simple shear conditions, frequently exhibit S-C fabric delineating the shear foliations. These foliations are attributed to the accumulation of finite strain (S-surfaces) and the localizartion of shear strain (C-surfaces); e.g^105^. At the initial stage of shearing, the angle between S and C is about 45° and by ongoing deformation it may reach 0°. However, such ideal shear zones are not observed in the concerned ZKB, as shearing and thrusting are intimately related in most outcrops. Shearing (strike-slip) and thrusting (oblique-slip) components can easily be deducted from subhorizontal and subvertical view of the same exposure. The oblique-lineation along thrust planes reflects an oblique-slip thrusting rather than pure-thrusting in the area (Fig. 10a). Stretching lineations and polymictic Hammamat conglomerates recorded along shear zones are also oblique and sometimes curved (Figs. 10b and 11). The prominent thrusting and shearing in the majority of the ZKB gave a robust impression that transcurrent shearing was a crucial compenent in what we can consider as shear-thrust tectonic setting. This makes this belt an ideal site to have a look for the shear zones accompanying to the thrusts and at the same time how large-scale thrust-sense shear zones evolve either in the central or southern parts of the ENS or elsewhere.

### Evidence of transpressional structuring

Transpression, the term introduced by^106^ to describe concurrent transcurrent shearing and contraction, was regarded later on by many workers; e.g^10,21,23,97,101^. as an important tectonic scenario in provinces of oblique convergence. Structural evidence for transpression have been documented in some orogenic belts, such as Zagros Orogen; e.g^107–109^. and East African Orogen; e.g^23,110,111^. Our field investigation documentd transpressional regime and approved its significat role in structural shaping of the ZKB. Inspection of the meso-scale shearing directions reveal that some of them combined with subparallel thrusting, demonstrating positive flower structures (see Fig. 6a, b). Asymmetric folds encountered inside the shear zones clarify the strike-slip component and the dip-slip component. Also, the geometry of the foliations and the obliquity of the stretching lineations (in mylonites) within the ENE- and NW-oriented shear zones reflect that the deformation is consistent with oblique dextral- and sinistral transpression; respectively. Besides, a wide variety of shear sense indicators recorded at the microscopic-scale, such as shear bands, mica fish and mineral fish, approve transpression.

### Proposed models for the evolution of Wadi Kareim structures

**Fig. 12:**
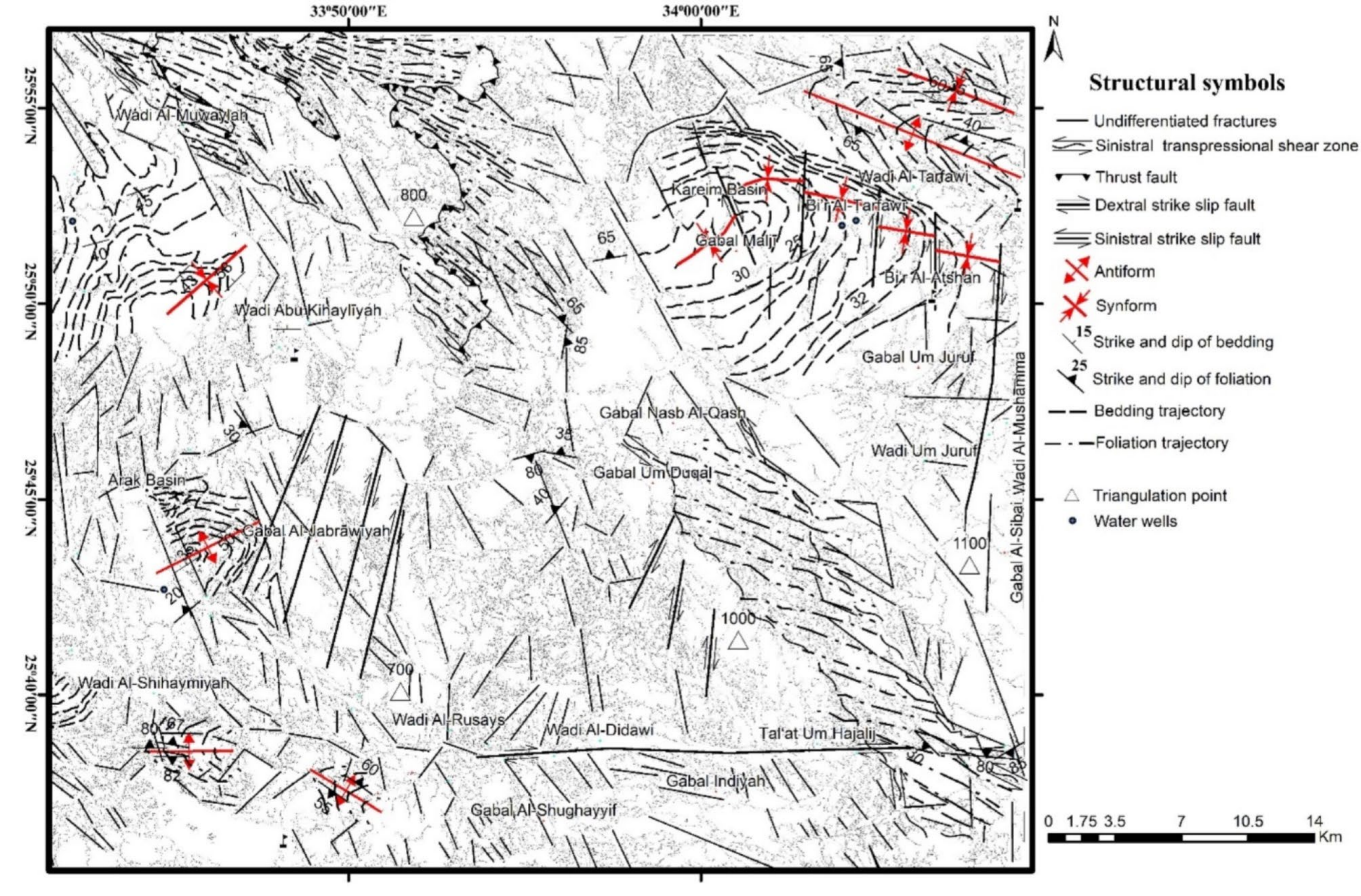
Structural map of the study area. Map created by ArcGIS Desktop v 10.7.1. https://www.esri.com/en-us/arcgis/products/arcgis-desktop/overview.

**Fig. 13:**
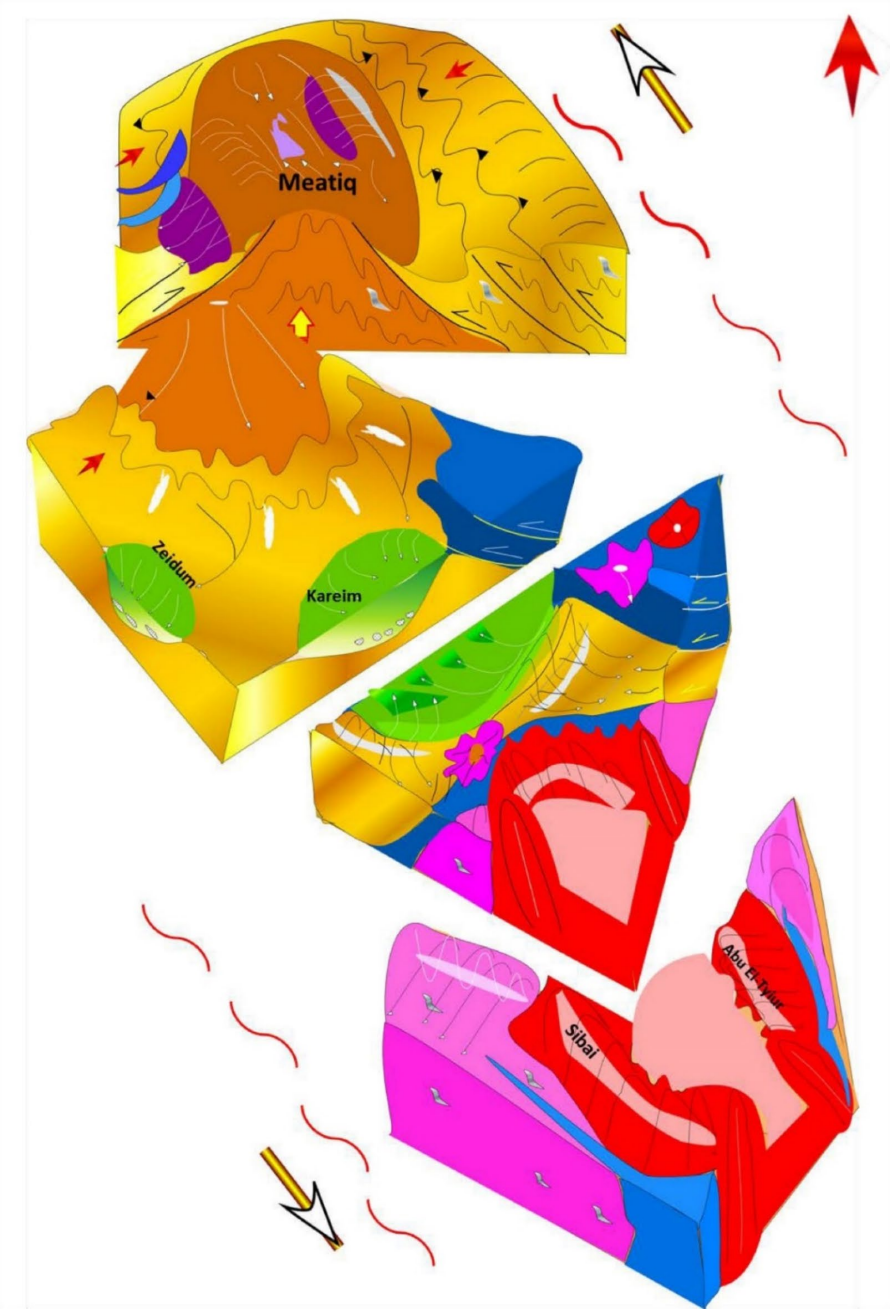
Proposed tectonic model for the ZKB. Block diagram created by CorelDRAW Standard 2024. https://www.coreldraw.com/en/product/coreldraw/standard/.

It was not an easy task to explain how all previously mentioned structures were formed, nor to propose a tectonic model for their formation. This is simply because, these structures (Fig. 12) exhibit a wide range of characteristics and therefore we show that no single model or interpretation can apply for all of them. However, Fig. 13 displays a proposed model illustrating the tectonic evolution of the area. The concerned area lies between Meatiq and Sibai domes. The deformation in the area encompasses three main stages: (1) the ENE-WSW obique convergence and the exhumation of the two domes, (2) the NW-SW Najd-related transcurrent shearing, and (3) the deposition of the Hammamat Volcanosedimentary Sequence in shear zone-related basins (Kareim and Zeidun Basins). The final stage of deformation was dominated by a transpressional regime, indicating that the structural evolution of the area took place under sinistral transpression. It is obvious that the three stages are genetically- and kinematically-related.

## Conclusions

The ZKB is one of the prominent belt in the Central Tectonic Province of the ENS (northwestern ANS). Our study fully constraints its spathio-temporal tectonic evolution. We conclude that the deformation in the ZKB involves three main stages. The oldest stage was concurrent with ENE-WSE oblique converence between East and West Gondwanalands. Exhumation of eye-catching Meatiq and Sibai domes (or core complexes? ) existed further north and south of the mapped area is affiliated to this stage. The second stage resulted from the the NW-SW transcurrent shearing affected the whole Najd-shear corridor in the ENS. Shearing paved the way to the formation of the two main basins (Kareim and Zeidun) where a remarkable volcanosedimentary sequence (Hammamat Group) deposited under transpressional regime during the last stage of deformation.

## Data Availability

The datasets used and/or analyzed during the current study are available from the corresponding author upon reasonable request.
